# RETRACTION: The Impact of Breast‐Conserving Surgery and Modified Radical Mastectomy on Postoperative Wound Complications in Patients With Early Breast Cancer

**DOI:** 10.1111/iwj.70577

**Published:** 2025-04-18

## Abstract

**RETRACTION:** YangX. , LinQ. , and WangQ. , “The Impact of Breast‐Conserving Surgery and Modified Radical Mastectomy on Postoperative Wound Complications in Patients With Early Breast Cancer,” International Wound Journal 21, no. 2 (2024): e14685, 10.1111/iwj.14685.PMC1085061242053028

The above article, published online on 07 February 2024, in Wiley Online Library (http://onlinelibrary.wiley.com/), has been retracted by agreement between the journal Editor in Chief, Professor Keith Harding; and John Wiley & Sons Ltd. Following an investigation by the publisher, all parties have concluded that this article was accepted solely on the basis of a compromised peer review process. The editors have therefore decided to retract the article. The authors did not respond to our notice regarding the retraction.

**RETRACTION:** X. Yang , Q. Lin , and Q. Wang , “The Impact of Breast‐Conserving Surgery and Modified Radical Mastectomy on Postoperative Wound Complications in Patients With Early Breast Cancer,” International Wound Journal 21, no. 2 (2024): e14685, 10.1111/iwj.14685.PMC1085061242053028

The above article, published online on 07 February 2024, in Wiley Online Library (http://onlinelibrary.wiley.com/), has been retracted by agreement between the journal Editor in Chief, Professor Keith Harding; and John Wiley & Sons Ltd. Following an investigation by the publisher, all parties have concluded that this article was accepted solely on the basis of a compromised peer review process. The editors have therefore decided to retract the article. The authors did not respond to our notice regarding the retraction.

